# Global status of Middle East respiratory syndrome coronavirus in dromedary camels: a systematic review

**DOI:** 10.1017/S095026881800345X

**Published:** 2019-02-21

**Authors:** R. S. Sikkema, E. A. B. A. Farag, Mazharul Islam, Muzzamil Atta, C. B. E. M. Reusken, Mohd M. Al-Hajri, M. P. G. Koopmans

**Affiliations:** 1 Department of Viroscience, Erasmus University Medical Center, Rotterdam, The Netherlands; 2 Ministry of Public of Health, Doha, Qatar; 3 Department of Animal Resources, Ministry of Municipality and Environment, Doha, Qatar

**Keywords:** Animal pathogens, coronavirus, emerging infections, zoonoses

## Abstract

Dromedary camels have been shown to be the main reservoir for human Middle East respiratory syndrome (MERS) infections. This systematic review aims to compile and analyse all published data on MERS-coronavirus (CoV) in the global camel population to provide an overview of current knowledge on the distribution, spread and risk factors of infections in dromedary camels. We included original research articles containing laboratory evidence of MERS-CoV infections in dromedary camels in the field from 2013 to April 2018. In general, camels only show minor clinical signs of disease after being infected with MERS-CoV. Serological evidence of MERS-CoV in camels has been found in 20 countries, with molecular evidence for virus circulation in 13 countries. The seroprevalence of MERS-CoV antibodies increases with age in camels, while the prevalence of viral shedding as determined by MERS-CoV RNA detection in nasal swabs decreases. In several studies, camels that were sampled at animal markets or quarantine facilities were seropositive more often than camels at farms as well as imported camels *vs.* locally bred camels. Some studies show a relatively higher seroprevalence and viral detection during the cooler winter months. Knowledge of the animal reservoir of MERS-CoV is essential to develop intervention and control measures to prevent human infections.

## Introduction

Middle East respiratory syndrome (MERS) is a highly fatal respiratory tract disease in humans that was first detected in 2012 in the Kingdom of Saudi Arabia (KSA) [1]. After its first detection, MERS-coronavirus (MERS-CoV) was being reported in human patients across the Arabian Peninsula, with occasional travel-related cases in other continents. As of the end of March 2018, a total of 2189 human laboratory-confirmed cases from 27 countries have been reported to the World Health Organisation (WHO), including 782 associated deaths [2]. Dromedary camels (*Camelus dromedaries*) have been shown to be the natural reservoir from where spill-over to humans can occur [3, 4]. Human-to-human infection is also reported frequently, especially in healthcare settings [5]. Sustained human-to-human transmission outside of hospital settings has not been shown yet [6]. Direct or indirect human contact with camels has resulted in repeated introductions of MERS-CoV into the human population [7]. It has been suggested that camels may have acquired MERS-CoV from a spill-over event from a bat reservoir, but evidence for that remains inconclusive [8]. Infections with MERS-CoV generally are thought to be mild or inapparent in camels [9], and are therefore of low economical or animal welfare significance.

This systematic review was done to compile and analyse all published data on MERS-CoV in the global camel population to provide an overview of current knowledge on the distribution, spread and risk factors of MERS-CoV infections in dromedary camels as a basis for the design of intervention and control measures to prevent human infections.

## Material and methods

On 2 May 2018, a literature search on PubMed was performed, using the terms ‘middle east respiratory syndrome coronavirus’ and ‘MERS-CoV’. Using the term ‘MERS’ did not result in any additional articles that fit the scope of this review. Only articles published in English were included. Two reviewers individually selected all original research articles containing laboratory evidence of MERS-CoV infections in dromedary camels in the field. Articles that were mentioned in Food and Agriculture Organization (FAO) updates [10] or in the references of included publications, but did not appear in the PubMed search were added. Subsequently, abstracts, follow-up studies of MERS-CoV-positive camels and genome studies without prevalence data were excluded from the analysis. Data on variables such as year of sampling, country, region, age, sex and animal origin were extracted and analysed. For each variable, the number of positive camels, total number of camels tested and the median percentage positivity was calculated. Data from experimental infection studies were not included in this analysis, but they were included in the review to provide additional information and context to the field studies. Additional information on the distribution and trade of dromedary camels was collected from references in the publications on MERS-CoV in camels and extracted from official FAO and World Organisation for Animal Health (OIE) databases [11, 12]. The additional literature on camel trade was collected in a less systematic way from PubMed.

## Results

### Literature search

**Fig. 1.**
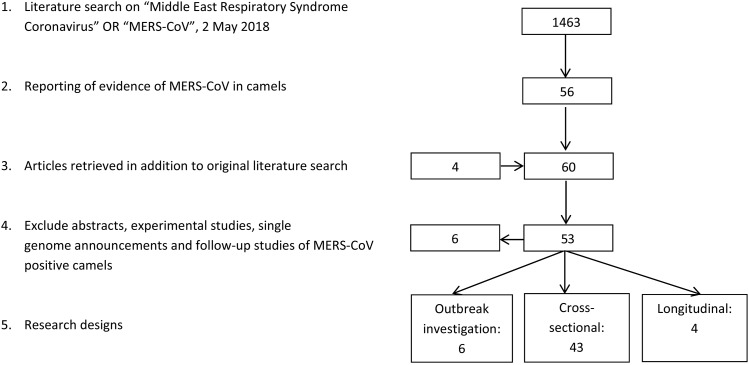
Results literature search.

The literature search resulted in a total of 53 papers (Fig. 1). Forty-three research papers described the results of cross-sectional studies in dromedary camel populations, six papers described outbreak investigations, including an analysis of camel samples, and four papers described longitudinal studies. In total, 33 papers describe camel studies in the Middle East, 13 studies investigated camels from Africa and the remaining seven surveys were from Spain, Australia, Japan, Bangladesh and Pakistan (Table 1).

**Table 1.** Summary table of included papers

References	Study design	Country of origin	Year	MERS-CoV RNA presence	MERS-CoV seroprevalence	Sex	Age	Imported/local	Sampling location	Other animals tested
Hemida *et al*. [50]	Cross-sectional	KSA	2010–2013		ppNT: 90% (280/310)		<1Y: 72% (47/65) 1–3Y: 95% (101/106) 4–5Y: 97% (74/76) >5Y: 92% (58/63)			Sheep 0% (0/100) Goat 0% (0/45) Chicken 0% (0/240) Cattle 0% (0/50)
Perera *et al*. [48]	Cross-sectional	Egypt	2013		MN: 98% (108/110)				Abattoir	Goat 0% (0/13) Sheep 0% (0/5) Buffalo 0% (0/8) Cattle 0% (0/25) Swine 0% (0/260) Wild birds (Hong Kong) 0% (0/204)
Reusken *et al*. [4]	Cross-sectional	Oman Spain (Canary islands)	2013 2012–2013		pMA: 100% (50/50) 14% (15/105)	Female: 100% (50/50) Male: 4% (2/50) Female:13% (7/55)	8–12Y: 100% (50/50)	Local Morocco: 0% (0/3)	Breeding farm Tourist farm	Bactrian camel 0% (0/4) Alpaca 0% (0/24) Llama 0% (0/7) Guanaco 0% (0/2) Cattle 0% (0/40) Goat 0% (0/120) Sheep 0% (0/40)
Reusken *et al*. [51]	Cross-sectional	Jordan	2013	Faecal: 0% (0/11)	pMA: 100% (11/11)	Male: 100% (11/11)	3–14m: 100% (11/11)			Sheep: 0% PCR (0/126) pMA: 5% (6/126): 0% (0/126) Cattle: PCR 0% (0/91) pMA: 0% (0/91) Goat: pMA/0% (0/150)
Alagaili *et al*. [31]	Cross-sectional	KSA	1992 1993 1994 1996 2004 2009 2010 2013	Nasal: 25% (51/202)	ELISA: 100% (1/1) 100% (2/2) 93% (114/123) 100% (6/6) 100% (6/6) 78% (64/82) 84% (37/44) 74% (150/203)		<2Y: 52% (50/96) 2–5Y: 88% (29/33) >5Y: 98% (54/55)			Goat: PCR 0% (0/36) ELISA 0% (0/35) Sheep: PCR 0% (0/78) ELISA 0% (0/112)
Alexandersen *et al*. [49]	Cross-sectional	UAE USA and Canada	2005 2000–2001		VNT/ELISA: 82% (9/11) 0% (0/6)	Male: 50% (2/4) Female: 100% (7/7)				Sheep 0% (0/20) Horse 0% (0/3)
Azhar *et al*. [66] Memish *et al*. [67]	Human outbreak investigation	KSA	2013	Nasal: 11% (1/9) Milk, urine, rectal: 0% (0/11)	IFA/ELISA: 100% (9/9)		<1Y: PCR 33% (1/3) IFA/ELISA: 100% (3/3) 2–5Y: IFA/ELISA 100% (1/1) >5Y: IFA/ELISA 100% (5/5)		Farm	
Chu *et al*. [9]	(Multiple) cross-sectional	Egypt	2014	Nasal: 4% (4/93) Nasal: 0% (0/17)	ppNT: 92% (48/52)		>6Y: 92% (48/52)	Sudan or Ethiopia Local	Abattoir Farm	
Corman *et al*. [36]	Cross-sectional	Kenya	Total 1992 1996 1998 1999 2000 2007 2008 2013		ELISA, total: 30% (228/774) 5% (1/22) 5% (2/37) 3% (2/62) 27% (71/266) 32% (82/258) 0% (0/28) 56% (103/183) 17% (8/47)		Adult: 37% (226/70) Juvenile: 25% (15/59)	Pakistan Local Local Local Local Local Local Local	Total, farm: 9% (40/436) Total, nomadic: 52% (229/439) Farm Farm Farm: 0% (0/50) Nomadic: 17% (2/12) Farm: 18% (32/175) Nomadic: 43% (39/91) Farm: 4% (4/112) Nomadic: 53% (78/146) Isolated Nomadic Farm: 3% (1/40) Nomadic: 100% (7/7)	
Haagmans *et al*. [3]	Human outbreak investigation	Qatar	2013	Nasal: 86% (12/14) Oral: 0% (0/14) Rectal: 0% (0/19)	IFA/VNT: 100% (14/14)				Farm	
Hemida *et al*. [19]	Longitudinal	KSA	2013–2014	Nasal: 33% (9/27) Oral: 0% (0/17) Rectal: 3% (1/37)			<2Y: 39% (7/18) 6–14Y: 22% (2/9)		Farm	
Hemida *et al*. [68]	Cross-sectional	KSA Australia Egypt	1993 2014 2014		ppNT: 90% (118/131) ppNT: 0% (0/25) ppNT: 100% (7/7)				Farm Farm and abattoir (feral) Abattoir	
Meyer *et al*. [37]	Cross-sectional	UAE	2003 2013		IFA: 100% (151/151) IFA: 96% (481/500)		>2Y: 100% (151/151) 2–8Y: 89% (89/100) >2Y: 89% (89/100)	KSA, Sudan, Pakistan and Oman UAE	Farm (racing): 89% (89/100) Farm (livestock camels): 100% (217/218) Isolated: 0% (0/5)	Bactrian camel 0% (0/16)
Muller *et al*. [32]	Cross-sectional	Somalia Sudan Egypt	1983–1984 1983 1997		ELISA: 84% (72/86) mNT: 81% (70/86) ELISA: 84% (159/189) mNT: 81% (153/189) ELISA: 81% (35/43) mNT: 79% (34/43)	Female: ELISA 84% (159/189)	>6Y: 84% (159/189)		Abattoir Farm	
Nowotny *et al*. [69]	Cross-sectional	Oman	2013	Nasal: 7% (5/76)						
Raj *et al*. [70]	Cross-sectional	Qatar	2014	Nasal: 2% (1/53)						
Reusken *et al*. [28]	Cross-sectional	Qatar	2013	Nasal: 15% (5/33) Rectal: 9% (3/33) Milk: 15% (5/33)	pMA: 100% (33/33) *Milk: pMA 100% (12/12), 75% (9/12)*	Female: 15% (5/33)	>5Y: PCR 42% (5/12) ELISA 100% (12/12)		Farm	
Reusken *et al*. [28]	Cross-sectional	Nigeria Tunisia Ethiopia	2010–2011 2009 2010–2011		pMA: 28% (100/358) pMA: 49% (99/204) pMA: 96% (181/188)		4–15Y: 28% (100/358) ⩽2Y: 30% (14/46) >2Y: 54% (85/158) ⩽2Y: 94% (29/31) >2Y: 97% (152/157)	Abattoir also serves Chad, Niger, CAR	Abattoir Farm	
Woo *et al*. [25]	Cross-sectional	UAE	2013	Faecal: 5% (14/293)	WB: 98% (58/59) IFA: 100% (59/59)		<1Y: PCR 21% (13/61): 98% (54/55) ⩾1Y: PCR: 0% (1/232): 100% (4/4)		Farm	
Al Hammadi *et al*. [71]	Human outbreak investigation	UAE	2015	Nasal: 100% (8/8)	ppNT: 100% (5/5)	Female: 100% (5/5)	<1Y: 100% (4/4) 10Y: 100% (1/1)	Oman	Border screening	
Chu *et al*. [72]	Cross-sectional	Nigeria	2015	Nasal: 11% (14/132)	ppNT: 95% (125/131)		>6Y: 95% (125/131)		Abattoir	
Crameri *et al*. [58]	Cross-sectional	Australia	2013–2014		VNT: 0% (0/307)				Abattoir: 231 Feral camel muster: 76	
Deem *et al*. [40]	Cross-sectional	Kenya	2013		pMA: 50% (166/335)		<6m: 36% (22/61) 6m–2Y: 30% (24/80) >2Y: 62% (120/194)		Farm: 48% (124/261) Nomadic: 57% (42/74)	
Farag *et al*. [26]	Cross-sectional	Qatar	2014	Nasal: 60% (61/101) Oral: 23% (23/102) Rectal: 15% (15/103) Bronchial: 7% (7/101) Lymph nodes: 9% (5/53)	pMA: 97% (100/103)		<1Y: PCR: 68% (50/73) ⩾1Y: PCR: 39% (11/28)		Abattoir	
Gutierrez *et al*. [33]	Cross-sectional	Canary Islands	2015		ELISA: 4% (7/170)	Male: 0% (0/101) Female: 10% (7/69)	⩾2Y: 4% (7/170) *All positives were aged 20–26Y*	African: 41% (7/17) Local: 0% (0/153)	Farm	
Khalafalla *et al*. [20]	Longitudinal	KSA	2013–2014	Nasal: 29% (28/96) Lung tissue 62% (56/91)			<4Y: 42% (15/36) ⩾4Y: 22% (13/60)		Abattoir, live animal market, veterinary hospital	
Shirato *et al*. [47]	Cross-sectional	Japan	2015	Nasal: 0% (0/4) Rectal: 0% (0/18) Oral: 0% (0/10)	ELISA: 0% (0/5)	Male: nasal PCR 0% (0/1) 0% (0/1) Female: nasal PCR 0% (0/3) 0% (0/4)	<2Y: 0% (0/1) >5Y: PCR 0% (0/3) 0% (0/3)		Zoo	Bactrian camels: PCR: 0% (0/6) ELISA: 0% (0/6)
Wernery *et al*. [55]	Cross-sectional	UAE	2015	Nasal: 0% (0/254) Milk: 0% (0/1333)	ELISA: 92% (234/254)	Female: ELISA 99% (132/133)	0–3m: ELISA: 75% (24/32) 4m: ELISA: 79% (11/14) 5–6m: ELISA: 89% (41/46) 7–12m: ELISA: 90% (26/29) >12m: ELISA: 99% (132/133)		Farm	
Wernery *et al*. [55]	Cross-sectional	UAE	2015	Nasal: 5% (45/871)	ELISA: 93% (786/843)		<1Y: PCR: 35% (24/68) ELISA 85% (92/108) 2–4Y: PCR: 3% (10/344) ELISA 97% (328/340) >4Y: PCR: 0% (0/250) ELISA 96% (298/310)		Farm	
Yusof *et al*. [73]	Cross-sectional	UAE	2014	Nasal: 2% (126/7803)				KSA Oman	Border screening: 2% (70/4617) Border screening: 1% (31/2853) Abattoir: 8% (25/303) Public escort and zoo: 0% (0/30)	
Meyer *et al*. [30]	Longitudinal 11 calf-dam pairs	UAE	2014–2015	At 6m (nasal): 18% (2/11) of calves, no dams	At day 0: MN/ELISA 0% (0/11) Maternal Ab peak at day 7 At 5–6m: 45% (5/11) At 12m: 100% (22/22)		Dams: ELISA: 100% (11/11)		Farm	
Miguel *et al*. [46]	Cross-sectional	Kazakhstan	2015		ppNT: 0% (0/455)	Female: 0% (0/455)			Farm	Bactrian camels: ppNT: 0% (0/95)
Muhairi *et al*. [29]	Human outbreak investigation	UAE	2014	Farms MERS patients (*n* = 2): Nasal: 10% (15/155) Surrounding farms: Nasal: 3% (27/992)					Farm	Sheep: 0% (0/34)
Sabir *et al*. [22]	Cross-sectional	KSA	2014–2015	Nasal: 12% (159/1309) Rectal: 0% (0/304)			⩽6m:15% (28/190) 6m–1Y: 18% (58/315) 1–2Y: 8% (42/509) 2–4Y: 10% (20/206) >4Y: 11% (5/46)	Local: 15% (133/893) Sudan: 6% (7/116) Somalia: 7% (19/291)	Abattoir: 0% (0/14) Farm: 11% (14/133) Market: 12% (145/1162)	
Al Salihi *et al*. [74]	Cross-sectional	Iraq	2015–2016	15% (15/100) (94 nasal, 6 oropharyngeal swabs)		Male: 18% (3/17) Female: 14% (12/83)	<1Y: 0% (0/9) 1–5Y: 15% (6/41) 5–10Y: 16% (6/38) >10Y: 25% (3/12)		Farm: 16% (13/80) Abattoir: 10% (2/20)	
Ali *et al*. [17]	Cross-sectional	Egypt	2014–2016	Nasal: 15% (435/2825) Rectal: 15% (18/114) Milk: 6% (12/187) Urine: 0% (0/26)	MN: 71% (1808/2541) *Milk: 20% (38/187)*	Male: PCR 21% (300/1439) MN: 72% (905/1254) Female: PCR 11% (115/1089) MN 66% (724/1090)	<2Y: PCR 16% (97/591) MN 37% (221/596) >2Y: PCR 10% (228/2234) MN 82% (1587/1945)	Local: PCR 12% (192/1658) MN 61% (1015/1655) Sudan, Somalia and Ethiopia: PCR 21% (243/1167) MN 90% (793/886)	Market: PCR 2.5% (4/159) MN 92% (159/172) Nomadic: PCR 1% (3/282) MN 72% (202/282) Farm: PCR 14% (189/1376) MN 59% (813/1373) Quarantine: PCR 36% (153/424) MN 95% (342/361) Abattoir: PCR 15% (86/584) MN 83% (292/353)	
Ali *et al*. [27]	Cross-sectional	Egypt	2014–2015	Nasal: 4% (41/1078)	MN: 84% (871/1031)	Male: PCR 3% (21/798) MN 85% (651/765) Female: PCR 7% (20/280) MN 83% (220/266)	⩽2Y: PCR 2% (2/82) MN 52% (42/81) >2Y: PCR 4% (39/996) MN 87% (829/950)	Local: PCR 1% (2/340) MN 76% (257/339) East Africa: PCR 3% (4/115) MN 72% (71/98) Sudan: PCR 6% (35/623) MN 91% (543/594)	Market: PCR 3% (9/290) MN 94% (273/289) Village: PCR 1% (2/340) MN 76% (256/339) Quarantine: PCR 2% (4/164) MN 96% (1557/164) Abattoir: PCR 9% (26/284) MN 77% (184/239)	Cattle: PCR 0% (0/35) MN 0% (0/35) Sheep: PCR 0% (0/51) MN 2% (1/51) Goat: PCR 0% (0/36) MN 0% (0/36) Buffalo: PCR 0% (0/4) MN 0% (0/4) Donkey: PCR 0% (0/15) MN 0% (0/15) Horse: PCR 0% (0/4) MN 0% (0/4) Bat: 0% (0/91)
Doremalen *et al*. [23]	Cross-sectional	Jordan	2016	Nasal: 67% (28/42) Rectal: 0% (0/42) Urogenital: 0% (0/42)	ELISA 82% (37/45)		<1Y: PCR 61% (11/18) ELISA 78% (14/18) 1–2Y: PCR 92% (12/13) ELISA 69% (9/13) 2–5Y: PCR 50% (5/10) ELISA 100% (10/10) >5Y: PCR 0% (0/1) ELISA 100% (4/4)		Farm PCR 77% (17/22) ELISA 77% (17/22) Nomadic: PCR (10/20) ELISA 87% (20/23)	Cattle: ELISA 0% (0/5) Sheep: ELISA 0% (0/10)
Falzarano *et al*. [53]	Cross-sectional	Mali	2009–2010		ELISA: 88% (502/571)	Male: 86% (210/245) Female: 92% (302/328)	1–2Y: 83% 3–8Y: 91% 9–16Y: 88%		Farm	Cattle and sheep: 0% (0/10)
Hemida *et al*. [24]	Longitudinal	KSA	2014–2015	Nasal: 4% (3/70) Rectal: 0% (0/70)	ppNT: 100% (70/70)		⩽2Y: 19% (3/16) >2Y: 0% (0/39)		Farm	
Kasem *et al*. [38]	Human outbreak investigation	KSA	2014–2016	Nasal: 10% (75/780) (camels with MERS patients contact)	ELISA: 71% (422/595)	Male: PCR 20% (49/245) ELISA 84% (127/152) Female: PCR 5% (26/535) ELISA 67% (295/443)	⩽2Y: PCR 15% (46/298) ELISA 57% (145/251) 2–4Y: PCR 6% (13/202) ELISA 79% (120/156) 4–6Y: PCR 4% (6/144) ELISA 81% (79/98) >6Y: PCR 7% (10/136) ELISA 87% (78/90)		Farm	
Miguel *et al*. [39]	Cross-sectional	Burkina Faso Ethiopia Morocco	2015	Nasal: 5% (27/525) Nasal: 11% (70/632) Nasal: 1% (5/343)	ppNT: 80% (421/525) 95% (600/632) 77% (265/343)	Seropositivity and CR-positive rate higher in females	Seropositivity rates increased, MERS RNA detection rate decreased with age			
Munyua *et al*. [75]	Cross-sectional	Kenya	2013		ELISA 90% (789/877)	Male: 81% (173/213) Female 93% (616/664)	1–4Y: 73% (209/285) 4–6Y: 99% (116/117) >6Y: 98% (466/476)		Farm: 71% (10/14) Nomadic: 91% (698/771)	
Saqib *et al*. [35]	Cross-sectional	Pakistan	2012–2015		ELISA: 56% (315/565) MN: 39% (223/565)	Male: ELISA/MN: 44% (96/217) Female: ELISA/MN: 36% (127/348)	⩽2Y: MN 29% (26/89) 2–5Y: 30% (62/208) 5–10Y: 51% (92/180) >10Y: 49% (43/88)			
Yusof *et al*. [41] Li *et al*. [76]	Cross-sectional	UAE	2015	Nasal: 29% (109/376)		Male: 27% (73/269) Female: 31% (33/107)	<1Y: 32% (81/255) >1Y: 21% (25/121)	Local: 25% (53/210) Oman: 50% (53/106): 5% (3/60)	Market	
David *et al*. [43]		Israel	2012–2017 (serum) 2015–2017 (nasal swab)	Nasal: 0% (0/540)	VNT: 62% (254/411)	Male: PCR 0% (0/54) Female: PCR: 0% (0/486)			Farm	Llama PCR 0% (0/19) ELISA: 37% (7/19) VNT: 32% (6/19) Alpaca PCR 0% (0/102) ELISA 34% (35/102) VNT: 32% (30/102)
Chu *et al*. [65]	Cross-sectional	Ethiopia	2016–2017	Nasal: 5% (5/102)						
Harrath *et al*. [77]	Cross-sectional	KSA	2016		ELISA: 84% (144/171)	Male: 83% (77/93) Female: 87% (68/78)	<2Y: 93% (66/71) 2–5Y: 78% (78/100)	Local	Farm	
Islam *et al*. [34]	Cross-sectional	Bangladesh	2015	Nasal: 0% (0/55)	ELISA/ppNT: 31% (17/55)	Male: ppNT 34% (10/29) Female: ppNT 27% (7/26)	<2Y: ELISA/ppNT 9% (1/11) ⩾2: ELISA/ppNT 36% (16/44)	Local: ELISA/ppNT 4% (1/24) India: ELISA/ppNT 52% (16/31)	Market: 63% (12/19) Farm: 14% (5/36)	Sheep: PCR 0% (0/18) ELISA/ppNT 0% (0/18)
Kasem *et al*. [78]	Cross-sectional	KSA	2015–2017	Nasal: 56% (394/698)			<2Y: 72% (303/423) >2Y: 33% (91/275)		Market: 42% (184/435) Abattoir: 80% (210/263)	

### Distribution and trade of camels

Most recent FAO statistics estimate the world population of camel to be around 29 million [11], of which approximately 95% are dromedary camels [13]. However, it is believed that the true population size is even larger due to inaccurate statistics and feral camels, such as the feral dromedary camel population in Australia that is estimated to be around 1 million [14]. Over 80% of the camel population lives in Africa. The main camel countries are Chad (6 400 000), Ethiopia (1 200 000), Kenya (2 986 057), Mali (1 028 700), Mauritania (1 379 417), Niger (1 698 110), Sudan (4 830 000), Somalia (7 100 000) and Pakistan (1 000 000) [12] (Table 2).

**Table 2.** Camel population and density

Country	Camel population (OIE, 2016)	Camel density (OIE, 2016) (*Animals per square kilometre*)
Africa		
Algeria	354 565 (OIE, 2014)	0.15 (OIE, 2014)
Burkino Faso	19 097	0.07
Djibouti	50 000	2.17
Egypt	66 233	0.07
Eritrea	385 283	3.18
Ethiopia	1 200 000	1.06
Kenya	2 986 057	5.12
Libya	110 000	0.06
Mali	1 028 700	0.83
Mauritania	1 379 417 (OIE, 2013)	1.34 (OIE, 2013)
Morocco	197 550 (OIE, 2014)	0.44 (OIE, 2014)
Niger	1 698 110 (OIE, 2013)	1.34 (OIE, 2013)
Nigeria	279 397	0.3
Sudan	4 830 000	1.93
Somalia	7 100 000	11.13
Chad	6 400 000	4.98
Tunisia	56 021	0.34
Middle East/Central Asia^a^		
Afghanistan	175 270	0.21
India^b^	400 000 (OIE, 2015)	0.12 (OIE, 2015)
Iran^b^	171 500	0.10
Iraq	81 205	0.19
Jordan	10 872 (OIE, 2014)	0.12 (OIE, 2014)
Kazakhstan^b^	170 513	0.06
Kuwait	80 790	4.53
Oman	257 713	1.21
Pakistan^b^	1 000 000	1.24
Qatar	77 417 (OIE, 2014)	6.77 (OIE, 2014)
Saudi Arabia	481 138	0.25
Syria	45 610	0.25
Turkmenistan^b^	122 900	0.25
UAE	392 667	4.74
Uzbekistan^b^	14 800	0.03
Yemen	459 366	0.87

a Excluding China and Mongolia because the large majority of camel population are Bactrian camels.

b Camel population exists of both dromedary and Bactrian camels[66].

A large number of camels are being transported from the Horn of Africa to the Middle East each year. These are mainly meat camels coming from the east of Africa going to Egypt, Libya and the Gulf states, and Sudanese camels that are being imported into the Middle East to participate in camel racing competitions [15]. For example, the FAO reported that Somalia exported 77 000 camels in 2014 [16]. The largest camel market in Africa is the Birqash market near Cairo (Egypt), where camels from Sudan and Ethiopia are most common, but trade routes include animals from Chad, Somalia, Eritrea and Kenya [17]. Imported camels are usually quarantined for 2–3 days at the border before they are allowed to enter Egypt [17]. Most Somali and Sudanese camels that are exported to the KSA are shipped from the ports of Berbera and Bosaso in North Somalia to the KSA ports of Jizan and Jeddah [15].

### Clinical and pathological features of MERS-CoV infections in dromedary camels

In general, only minor clinical signs of disease have been observed in animals infected with MERS-CoV and most MERS-CoV infections do not appear to cause any symptoms [9]. Disease symptoms that have been described after experimental and field infections are coughing and sneezing, respiratory discharge, fever and loss of appetite [18–20]. Although MERS-CoV RNA can be detected in several organs after experimental infection, in studies of natural infectious virus it has only been detected in the tissues of the upper and lower respiratory tract and regional lymph nodes of the respiratory system in part of the infected camels. Histologically, a mild-to-moderate inflammation and necrosis could also be seen on the upper and lower respiratory tract. No viral antigen or lesions were detected in the alveoli. Histopathological examination showed that the nasal respiratory epithelium is the principal site of MERS-CoV replication in camels [18, 21].

### Virus shedding and antibody response

In one study investigating experimental infection of camels, MERS-CoV shedding started 1–2 days post-infection (dpi). In that study, infectious virus could be detected until 7 dpi, and viral RNA until 35 dpi in nasal swab samples and, in lower amounts, in oral swab samples [18]. No infectious virus or viral RNA was detected in faecal or urine samples [18]. Viral RNA detection in nasal, but also rectal swabs of camels after experimental infection until day 14, has been confirmed in a recent vaccine study [21].

In the field surveys included in this review, MERS-CoV RNA has been described in rectal swab samples, although other field studies report negative results [3, 22–24] and when viral RNA can be detected, the positivity rate of rectal swabs is lower compared with nasal swab samples [19, 25–27]. Oral swabs are usually negative or show a lower positivity rate even when nasal swabs test positive for MERS-CoV RNA [3, 19, 26]. Some studies have reported MERS-CoV RNA in milk samples [27, 28]. Longitudinal studies of camel herds show that PCR results of nasal swabs can remain positive after 2 weeks [27, 29]. When an interval of sampling of 1 or 2 months was maintained, nasal swabs become negative for viral RNA in the next sampling round [24, 30].

MERS-CoV infections have also been detected in camels with MERS-CoV antibodies, both in calves with maternal antibodies as well as older camels that had already acquired antibodies from a previous infection. However, virus replication and thus the virus load is generally lower in infected seropositive animals compared with seronegative camels [19, 21, 23, 24, 30, 31].

Little is known about the longevity of antibody titres after infection from longitudinal studies. A study following camels on a closed farm found that neutralizing antibodies remained consistent during a year [30], while other studies found that antibody titres rapidly drop by 1–4-fold within a period often as short as 2 weeks [24, 27].

### Worldwide distribution of MERS-CoV in dromedary camels

**Fig. 2.**
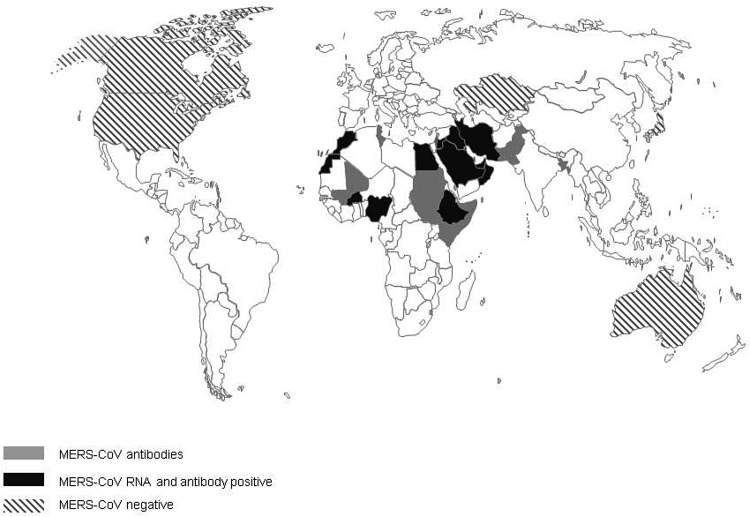
Virological and serological evidence for MERS CoV in dromedary camels.

The first evidence of MERS-CoV in camels described so far is the detection of antibodies to MERS-CoV in camel sera from Somalia and Sudan from 1983 of which 81% tested positive [32]. Additional serological evidence of the widespread presence of MERS-CoV infection in camels, included in this review, has been found in 18 additional countries: Bangladesh, Burkina Faso, Egypt, Ethiopia, Iraq, Israel, Jordan, Kenya, KSA, Mali, Morocco, Nigeria, Oman, Pakistan, Qatar, Spain, Tunisia and the UAE (Fig. 2). In addition, Promed mail reported that virus-positive camels had been found in Kuwait and Iran, the latter reportedly in imported animals (Archive number 20140612.2534919 and 20141029.2912385). In 11 countries, serological findings were complemented with the finding of viral RNA in dromedary camels: Burkina Faso, Egypt, Ethiopia, Iraq, Jordan, KSA, Morocco, Nigeria, Oman, Qatar and the UAE. Investigations of MERS-CoV circulation amongst dromedary camels in Australia, Japan, Kazakhstan, USA and Canada did not find any proof of MERS-CoV circulation. All countries where MERS-CoV circulates in the camel population, with the exception of Spain (Canary Islands), Pakistan and Bangladesh, are located in the Middle East or Africa [4, 33]. One out of 17 camels that had MERS-CoV antibodies in Bangladesh was born in Bangladesh, 16 others were imported from India [34]. However, there have not been any additional reports of MERS-CoV in camels in India. There is no record of foreign origin of the seropositive camels from Pakistan [35]. Moreover, in previous studies there had already been evidence of seropositive camels that originate from Pakistan [37, 58].

When combining serology data from all papers included in this review, the overall median seroprevalence of camels in Africa is 81% (6106/8526; range 28–98%), compared with a median seroprevalence of 93% (3230/3846; range 53–100%) in camels from the Middle East. Based on viral shedding studies from African countries, the median rate of viral shedding was 5% (1108/6318; range 1–15%), compared with 12% in camels from the Middle East (1191/14902; range 0–100%).

### Risk factors of MERS-CoV in dromedary camels

#### Age

The seroprevalence of MERS-CoV antibodies increases with age in camels, while the fraction of camels that test positive for MERS-CoV RNA in their nasal swabs decreases with age [17, 31, 36, 38, 39]. When all serological results of papers that included sufficient age information is combined, the median seroprevalence of camels aged under 2 years is 52% (992/1972; range 0–100%), while the age groups 2–5 years (702/924; range 30–100%) and over 5 years old (1226/1370; range 0–100%) had a combined median seroprevalence of 97%. In the virological studies reporting age breakdown, the median rate of nasal shedding in 0–2 years old camels was 34% (718/2612; range 0–100%) of cases, compared with 2% (91/1142; range 0–100%) in camels older than 2 years.

#### Sex

Some individual studies show a significantly higher seroprevalence in female camels compared with males [27, 39], while others show the opposite [38] or do not find any significant difference [17, 35]. Similar disagreeing results are published for the presence of MERS-CoV RNA in male *vs.* female camels [17, 27, 38, 39].

In the studies in this review where sex of camels was recorded, a total of 4810 serum samples from female camels and 3458 samples from male camels were collected and analysed for MERS-CoV antibodies, compared with 2007 *vs.* 2505 nasal swabs for viral RNA testing. Approximately three times more female camels were sampled at farms, while male camels were in the majority in studies that looked at MERS-CoV prevalence of camels at slaughterhouses, live animal markets and quarantine areas. The overall median seroprevalence of male and female camels in our review is 50% and 67%, respectively (range 0–100%; excluding results from Israel and Kazakhstan). The median percentage of presence of viral RNA is 18% in nasal swabs of male camels (range 0–21%) compared with 9% in female camels (range 0–100%), in our review.

#### Sampling location and herd characteristics

In several studies, camels that were sampled at animal markets or quarantine facilities were seropositive more often than camels at farms [17, 22, 27, 34]. Combining serological laboratory results of camels in our review with sufficient background information with regard to the sampling location does not result in the same pattern, with a median seroprevalence of 84% (5632/8115; range 0–100%; excluding Australia and Spain) in camels from farms and 80% (943/1005; range 28–98%) in the camel population sampled at markets and quarantine facilities. Studies in Egypt found a significantly higher PCR positivity rate in camels sampled in abattoirs or quarantine facilities, but these results could not be confirmed by other papers in this review [17, 27].

When comparing differences in seroprevalence or virus RNA-positive rate in nomadic *vs.* sedentary camel herds, some authors did not find a statistical difference between the two herd management types [39, 40], while others found some evidence of higher seroprevalences in nomadic herds [27, 36]. One study in Kenya looked at the differences between herds with different levels of isolation, and did not find significant differences in MERS-CoV antibody levels [40].

#### Animal origin

Most studies that compared local camels with imported camels suggested that imported camels are seropositive for MERS-CoV more often [9, 17, 27, 34, 41], although not all differences were significant.

Two studies in Egypt found a significantly higher RNA positivity rate in imported camels from East Africa compared with domestically bred camels [17, 27], while another study executed in the KSA found a significantly higher number of MERS-CoV RNA-positive results amongst local camels *vs.* camels from Sudan and Somalia [22].

#### Seasonal variation in MERS-CoV circulation in the camel population

Although MERS-CoV was detected almost year-round in camels, some studies show a relatively higher seroprevalence and viral detection during the cooler winter months [17, 20, 27, 38].

### MERS-CoV in non-dromedary animals

MERS-CoV antibodies have been detected in llamas and alpacas in Israel and in alpacas in Qatar [42, 43]. To date, no MERS-CoV antibodies or viral RNA have been detected in Bactrian camels [4, 37, 44–47] (Table 1 and Table 3). Swine, goats and horses that were included in the field surveys in our review all tested negative for MERS-CoV RNA and antibodies [4, 17, 31, 48–52]. MERS-CoV antibodies were detected in two studies in sheep in Egypt and Qatar, although in very low numbers [17, 51]. However, most surveys that investigated sheep did not find evidence of MERS-CoV infection or exposure [4, 23, 29, 31, 34, 48–51, 53].

**Table 3.** MERS-CoV in non-dromedary animals in the field

Species	Seroprevalence	Viral RNA^a^
Bactrian camel	0% (0/505) (Netherlands, Chile [4]; UAE [37]; Mongolia [44]; China [45]; Kazachstan [46]; Japan [47])	0/390 (China [45], Mongolia [44])
Alpaca	24% (30/126) (Israel(+) [43], Netherlands, Chile [4]) *100% (15/15), Qatar* [42]^b^	0% (0/102) (Israel [43]) *0% (0/15)*(*Qatar:* [42])^b^
Llama	23% (6/26) (Israel (+) [43], Netherlands, Chile [4])	0% (0/19) (Israel [43])
Guanaco	0% (0/2) (Chile [4])	–
Cattle and buffalos	0% (0/258) (KSA [50]; Egypt [27, 48]; The Netherlands [4]; Jordan [23, 51])	0% (0/35) (Egypt [27])
Swine	0% (0/260) (Egypt [48])	–
Sheep	0.2% (1/482)^c^ (KSA [31, 50]; Egypt (+) [27, 48], The Netherlands [4]; Jordan [23, 51]; UAE [29, 49]; Bangladesh [34])	0% (0/307) (Jordan [51]; KSA [31]; Egypt [27]; Bangladesh [34])
Goats	0% (0/399) (KSA [31, 50]; Egypt [27, 48]; Spain, The Netherlands [4]; Jordan [51])	0% (0/72) (KSA [31]; Egypt [27])
Horses, donkeys	0% (0/22) (Egypt [27]; UAE [49]) *0% (0/192)(UAE* [52])^b^	0% (0/19) (Egypt [27])
Birds	0% (0/444) (KSA [50]; HK [48])	–
Bats	0% (0/91) (Egypt [27])	

a MERS-CoV RNA in nasal swabs.

b Articles that were not included in the original literature search, because no camels were investigated in these studies.

c Six additional sera from sheep in Qatar tested positive by protein microarray (pMA), but could not be confirmed by NT.

## Discussion

The publications in this review show that the MERS-CoV mainly circulates in dromedary camel populations in the Middle East and part of Africa, and has been infecting dromedary camels in Africa for more than three decades. Antibodies have also been found in Arabic camel sera from the early 90s [31, 32]. However, MERS-CoV was discovered until 2012, after the first human cases appeared [1], which is probably due to the minor clinical symptoms of MERS-CoV infections in camels [18]. Most camel surveys were conducted in the Middle East and some northern and eastern African countries, but significant data gaps currently still exist in the north and west of Africa, in countries that have camel populations of 100 000 to more than a million animals, such as Algeria, Libya, Mauritania and Niger. Even less is known about the central Asian region. Some evidence of MERS-CoV circulation in camels of Pakistan and Bangladesh was recently published, but data is lacking from Afghanistan and India. Knowledge on the presence of MERS-CoV in the animal reservoir is a crucial first step to assess whether MERS-CoV could be a relevant public health threat in these regions.

MERS-CoV infections are mainly detected in calves and young camels [30, 31]. The research included in this review shows that the IgG positivity rate increases gradually in dromedary camels of increasing age while the MERS-CoV RNA detection rate decreases. Maternal IgG antibodies in camels are acquired through the intake of colostrum during the first 24 h post-parturition. After 24 h, antibody levels in the dam's milk decrease rapidly [54]. One study showed that maternal antibodies in calves peak at 7 days post-parturition and decline in the following 6 months. After 5–6 months, over half of the calves did not have maternal neutralizing antibodies in their serum any longer [30]. However, in other field studies, the titre of MERS-CoV-specific antibodies is still low at 1 month of age and increases with age in dromedary calves [27, 55]. A lower or undetectable antibody levels in young camels is likely to explain the higher MERS-CoV RNA detection rate. In adult camels, a much higher MERS-CoV seroprevalence can be found, which is probably due to a long-lasting immune response against a MERS-CoV infection or multiple re-infections with MERS-CoV. Immunity is not sterilizing, as MERS-CoV infection and shedding have also been shown in adult camels that have MERS-CoV antibodies [19, 21, 23, 24, 30, 31].

Several articles have analysed seroprevalence and virus shedding data in relation to factors, other than age, that may explain differences in seroprevalence and MERS-CoV RNA-positive rate in camels, such as sex, sampling location, herd characteristics and animal origin. Our review shows that there is considerable heterogeneity in results. In addition, comparison between studies is difficult given the lack of standardisation of study designs. A key factor to consider when comparing studies is the difference in distribution of male and female camels amongst different disciplines of camel husbandry. Females are mainly used for milking and reproduction. As a result, they often stay at farms. Male camels, especially of young age (<1 year old), are the predominant sex in slaughterhouses and amongst camels used for transport [39, 56]. This also influences the risk profile of acquiring a MERS-CoV infection. Female camels are in closer contact with calves, who are more susceptible to infection and shed virus in higher quantities compared with older camels [30]. On the other hand, meat and transport camels (predominantly male) travel more, leading to increased contact with other camels and camel herds, and therefore a higher chance of exposure to MERS-CoV. Some papers in this review suggest that there is a generally lower infection rate of domestically bred camels and camels on farms compared with imported camels and camels on animal markets or in quarantine facilities. This may be explained by the same increased contact rate and mixing of camel herds, leading to an increased chance of MERS-CoV exposure and spread.

The increase in MERS-CoV circulation in winter and spring can have multiple explanations. Firstly, the winter is the calving season [10], which leads to a larger proportion of young animals that usually have a higher number of MERS-CoV infections and virus excretion. Moreover, in winter season, there is a major increase of camel and human movements due to camel racing competitions, camel breeding, trading and movements to grazing grounds, which increases the chance of virus spread. Additionally, cooler temperatures may facilitate coronavirus survival in the environment [57].

In experimental studies, llama's and alpaca's are shown to be susceptible to infection with MERS-CoV [58, 59], which was confirmed by two papers in our review, describing serologically positive llamas and alpacas in Israel and alpacas with MERS-CoV neutralizing antibodies in Qatar [42, 43]. In experimental settings, animal-to-animal transmission has been shown for alpacas, making them a possible risk population for human infections [58]. Two studies in our review also found anti-MERS-CoV antibodies in sheep [17, 51] but experimental inoculation of sheep did not result in MERS-CoV replication or antibody development [59, 60]. However, the DPP4 receptor, the entry receptor for MERS-CoV, is present in sheep tissues, making it possible for the virus to bind to the sheep respiratory tract which may explain the finding of MERS-CoV antibodies [61]. Pigs also express the DPP4 receptor in their respiratory tract, and viral replication in experimental settings has been shown for pigs, but no antibodies or MERS-CoV RNA have been found in pigs during field surveys [48, 59]. This may be explained by the limited viral shedding in pigs and the absence of animal-to-animal transmission [62, 63].

We show that dromedary camels are present in large parts of the African and Asian continent, and that MERS infections in dromedary camels are widespread. However, human infections due to spill-over from the dromedary camel reservoir have not been reported in Africa [10]. Several explanations for the difference in human cases between the Arabian Peninsula and Africa have been suggested, such as differences in cultural habits, camel husbandry, prevalence of comorbidities, under detection or genetic factors in the local population [64]. Moreover, West African viruses were found to be phylogenetically and phenotypically distinct from the MERS-CoV viruses that caused human disease in the Middle East [65].

Increased knowledge on the animal reservoir of MERS-CoV needs to be combined with research on MERS prevalence and risk factors in humans to assess the true public health risk. Moreover, the absence of human disease, combined with the mild symptoms in camels, caused by MERS, will likely have a negative effect on the willingness to implement interventions and the cost-effectiveness of possible interventions in some areas.

## Conclusion

Since the discovery of MERS-CoV in 2012, the dromedary camel has been identified as the animal reservoir of human infections with the MERS-CoV. However, the exact route of human primary infections is still unknown. Moreover, the scale of the spread and prevalence of MERS-CoV in the camel reservoir is not fully known yet since there is still a lack of MERS-CoV prevalence data in some countries that harbour a very significant proportion of the world camel population. However, knowledge of the animal reservoir of MERS-CoV is essential to develop intervention and control measures to prevent human infections. Prospective studies that include representative sampling of camels of different age groups and sex, within the different husbandry practices, are needed to fully understand the patterns of MERS-CoV circulation. Such studies are important as they may give more information on critical control points for interventions to reduce the circulation of MERS-CoV and/or exposure of humans.
