# Global status of Middle East respiratory syndrome coronavirus in dromedary camels: a systematic review – CORRIGENDUM

**DOI:** 10.1017/S0950268819000669

**Published:** 2019-05-17

**Authors:** R. S. Sikkema, E. A. B. A. Farag, Mazharul Islam, Muzzamil Atta, C. B. E. M. Reusken, Mohd M. Al-Hajri, M. P. G. Koopmans

Due to an oversight during the final editing of the manuscript, there has been a shift in the references of **table 1**. The editorial team apologises for this mistake.

- Azhar et al. [66] should be [67]
- Memish et al. [67] should be [68]
- Hemida et al. [68] should be [69]
- Nowotny et al. [69] should be [70]
- Raj et al. [70] should be [71]
- Al Hammadi et al. [71] should be [72]
- Chu et al. [72] should be [73]
- Yusof et al. [73] should be [74]
- Al Salihi et al. [74] should be [75]
- Munyua et al. [75] should be [76]
- Li et al. [76] should be [77]
- Harrath et al. [77] should be [78]
- Kasem et al. [78] should be [79]

Additionally, table 1 cites Crameri et al [1] but should have cited Crameri et al. [2], which was not present elsewhere in the paper.
